# L'Hôpital Cochin

**Published:** 1831-06

**Authors:** 


### L'HÔPITAL COCHIN.

### *General Emphysema, formed by a Combustible Gas.*

THE details of this very curious case were lately read by M. BALLY, at the Académie Royale de Médecine. A man, twenty-five years of age, who had been ill for fifteen days, was admitted into the hospital with symptoms of typhus fever. He also complained of severe pain in the left thigh, and, whilst he was in a state of delirium, he said he had been bitten on the knee by a dog. The limb was most attentively examined, but not the slightest trace of such an accident could be discovered. The thigh and scrotum were much swollen. He died the following day.

*Dissection, eight hours after death.* The surface of the body was soiled by blood which had also transuded through the integuments of the thighs: some blood had also been discharged from the nose. The whole body was emphysematous, but the left inferior extremity was in this state to a very high degree: it was double its natural size, of a brown colour, and covered with numerous phlyctenæ, some black, of great extent, and collected in clusters, from which escaped a reddish serous fluid, mingled with a quantity of gas; others were white, and from these nothing but air escaped. The limb sounded upon percussion, and, when it was pressed with the hand, crepitation was distinctly heard. The abdomen was much distended with gas. The face and temples were deeply injected with blood, and were of a violet colour. Upon dividing the scalp, a large quantity of dark-red blood escaped. The nerves and lungs presented no remarkable appearances. Heart pale and void of blood. In the intestines were observed those alterations which are so commonly detected in cases of typhus fever. Bubbles of air filled the vessels of the pia mater and the left vena saphena. The lymphatic ganglions of the mesentery were enlarged, and contained gas, which took fire from the flame of a taper, and produced an explosion. The same phenomena also followed the exit of air which was contained in the legs, thighs, and scrotum, when incisions had been made into these parts. A puncture was made into the abdomen, and the gas which escaped also took fire, and burned for some time: the flame was blue at its base, and white at its summit. The combustion extended to the puncture which had been made with a trocar. The edges of this aperture became black, and were consumed, and the aperture itself was enlarged to double the size it had originally been made. The gas which was contained in the subcutaneous cellular tissue of the thorax was equally inflammable.

M. Bally believes that this case is unparalleled in the records of science, and that it may throw some light on "spontaneous combustion." These combustions he conceives to depend upon the secretion of gas in the different tissues, by some morbid action, which gas must be inflamed by an electric spark from the living body. The inflammable gas which constituted the emphysematous swellings in the above case could not be considered as a post-mortem product, for emphysema existed before death. The body was opened eight hours after death, and presented no appearance of putrefaction.

The recital of this extraordinary case led to some discussion in the Academy. M. Breschet admitted that a vital morbid action might give rise to the production of a large quantity of inflammable gas, and that in this manner spontaneous combustion might originate, but he doubted whether the natural electricity of the body could inflame this gas. The contact of a body in a state of ignition is necessary, as has been the case in all the instances of spontaneous combustion which have been observed for some years,

Many cases were mentioned in which inflammable gas had been detected in different parts of the body. M. Deneux related an instance, which was witnessed by Leduc, in which there escaped from the uterus, during labour, a quantity of gas, which took fire and exploded. Other members related instances in which enormous emphysematous swellings had been very rapidly formed, both during life and after death. By some it was supposed that these gaseous productions arise from a morbid secretion; while others imagined that they are generally, if not always, the effect of a putrescent decomposition, which may commence before death, but which is usually posterior to it.

For much curious speculation concerning the probable causes of spontaneous combustion, together with the description of some interesting cases, we may refer to the 43d and 44th volumes of the Philosophical Transactions.

